# Utility and Usability of Two Forms of Supplemental Vibrotactile Kinesthetic Feedback for Enhancing Movement Accuracy and Efficiency in Goal-Directed Reaching

**DOI:** 10.3390/s23125455

**Published:** 2023-06-09

**Authors:** Ramsey K. Rayes, Rachel N. Mazorow, Leigh A. Mrotek, Robert A. Scheidt

**Affiliations:** 1 Joint Department of Biomedical Engineering, Marquette University and the Medical College of Wisconsin, Milwaukee, WI 53233, USA; rrayes@mcw.edu (R.K.R.); rachel.mazorow@marquette.edu (R.N.M.); leigh.mrotek@marquette.edu (L.A.M.); 2 Medical School, Medical College of Wisconsin, Milwaukee, WI 53226, USA

**Keywords:** sensory augmentation, joint angle encoding, cartesian endpoint encoding, hand kinematics, movement decomposition, user experience

## Abstract

Recent advances in wearable sensors and computing have made possible the development of novel sensory augmentation technologies that promise to enhance human motor performance and quality of life in a wide range of applications. We compared the objective utility and subjective user experience for two biologically inspired ways to encode movement-related information into supplemental feedback for the real-time control of goal-directed reaching in healthy, neurologically intact adults. One encoding scheme mimicked visual feedback encoding by converting real-time hand position in a Cartesian frame of reference into supplemental kinesthetic feedback provided by a vibrotactile display attached to the non-moving arm and hand. The other approach mimicked proprioceptive encoding by providing real-time arm joint angle information via the vibrotactile display. We found that both encoding schemes had objective utility in that after a brief training period, both forms of supplemental feedback promoted improved reach accuracy in the absence of concurrent visual feedback over performance levels achieved using proprioception alone. Cartesian encoding promoted greater reductions in target capture errors in the absence of visual feedback (Cartesian: 59% improvement; Joint Angle: 21% improvement). Accuracy gains promoted by both encoding schemes came at a cost in terms of temporal efficiency; target capture times were considerably longer (1.5 s longer) when reaching with supplemental kinesthetic feedback than without. Furthermore, neither encoding scheme yielded movements that were particularly smooth, although movements made with joint angle encoding were smoother than movements with Cartesian encoding. Participant responses on user experience surveys indicate that both encoding schemes were motivating and that both yielded passable user satisfaction scores. However, only Cartesian endpoint encoding was found to have passable usability; participants felt more competent using Cartesian encoding than joint angle encoding. These results are expected to inform future efforts to develop wearable technology to enhance the accuracy and efficiency of goal-directed actions using continuous supplemental kinesthetic feedback.

## 1. Introduction

When performing goal-directed actions, people typically integrate information from multiple senses (e.g., vision and proprioception) to control their body movements ([1,2,3,4]; but see [5]). Usually, multimodal sensory integration is performed without conscious thought ([6,7]; see also [8]). Sometimes, however, one of the senses may be unavailable in the short- or long-term (cf., [5]); visual attention might need to be diverted when reaching for a cup of coffee, or proprioception may be permanently impaired after a neuromotor injury such as stroke ([9]; see also [10]). The current study is based on the idea that it may be desirable in some cases to enhance the accuracy and efficiency of movements by providing continuous supplemental kinesthetic feedback of limb or tool motion in real-time using sensory augmentation techniques (c.f., [10,11,12,13,14,15,16,17,18,19]).

We envision a future where intelligent wearable technologies enhance functional movements by sensing the state of the body and its physical surroundings, synthesizing real-time sensory feedback optimized to achieve control goals, and delivering the feedback in an inconspicuous way that does not interfere with other critical behaviors. Recent advances in wearable sensors and computing have made possible the development of novel augmentation technologies that have potential applications in a wide array of fields, including aerospace [20,21], navigation [22], virtual reality [23], sports [24,25], and healthcare [26,27]. In healthy individuals, supplemental feedback has been used to augment motor performance in complex tasks, such as robot-assisted surgery [28,29,30], and to promote motor learning while playing an instrument or sports [31,32,33]. In individuals with sensorimotor deficits, vibrotactile stimuli have been applied to improve sensorimotor control by exciting corticospinal pathways contributing to the regulation of movement and/or reflex activity [34]. As such, efforts to enhance real-time feedback control of the arm and hand draw on a rich body of prior research.

The requirement that a sensory augmentation system be continuously wearable places strict constraints on the technology. Use of the system should minimize the potential for injury, be easy to don/doff, minimize demands on visual attention, not interfere with conversation, and be inconspicuous so that social stigmatization is minimized (c.f., [35,36,37]). There are several ways that supplemental sensory information can be delivered by a wearable system, including acoustic stimuli [38,39,40], visual stimuli [41], and electrotactile stimuli [42,43]; we propose that only vibrotactile stimulation readily satisfies all the criteria; electrotactile stimulation can cause skin breakdown [44] or interfere with speech [45], acoustic stimulation can interfere with perception of the spoken word, and visual augmentation places a high load on visual attention. Several stimulation sites proposed by Kaczmarek et al. [11] can be discounted if the wearable system is to be inconspicuous and easy for individuals to don and doff: these include the abdomen, back, fingertip, forehead, and tongue. By contrast, donning/doffing can be relatively easy for wearable technology applied to the proximal arm segments. Preliminary experimental studies have found that a supplemental vibrotactile feedback system consisting of two tactors per degree of freedom can be intuitive to use [10,17] and can promote degrees of movement accuracy and efficiency that meet or exceed those observed during movements guided by intrinsic proprioceptive feedback in the absence of visual feedback [18,19].

There are at least four ways to encode information relevant to the control of limb motion into supplemental vibrotactile feedback. These include continuous feedback of limb state relative to an arbitrary reference body configuration [17,18]; continuous error feedback relative to a goal [12,14,16,32,46]; continuous optimal feedback relative to some arbitrary cost function [47]; and intermittent alarms indicating undesirable conditions [48]. Regardless of which form the vibrotactile feedback takes, the cues must be designed and applied such that the encoded information can be easily perceived and interpreted by the user [49,50,51,52]. User experience research notes that the usefulness of a system derives from two main aspects of its design evaluated within a specific context. These are utility (the capacity of a system to fulfill a real user need) and usability (the capacity for users to readily understand and learn how to use the system). The immediate goal of the current study is to compare the objective utility and subjective user experience offered by two biologically inspired ways of encoding limb kinematic information into vibrotactile feedback for the real-time control of goal-directed movements. These include the encoding of hand position within a reference frame modeled in some respects after visual feedback (Cartesian Endpoint Encoding; CEE) and the encoding of arm configuration within a reference frame modeled after intrinsic proprioceptive feedback (Joint Angle Encoding; JAE).

We asked healthy human participants to hold the handle of a robotic manipulandum while making point-to-point, goal-directed reaches in the horizontal plane. We compared the utility of the two encoding schemes of supplemental kinesthetic feedback in enhancing the accuracy and efficiency of reaches performed in the absence of concurrent visual feedback. We also compared the extent to which the two encoding schemes could foster positive user experiences, which we assessed using standard surveys of system usability [53], intrinsic motivation [54], and user satisfaction [55,56]. We specifically tested the following hypotheses: (1) after a brief training period, supplemental vibrotactile feedback can improve reach accuracy in the absence of concurrent visual feedback for both encoding schemes; (2) when reaching without vision, one or the other encoding scheme will promote better reach accuracy and/or efficiency; and (3) that participants will prefer one encoding method over the other. The first two hypotheses were tested via analysis of kinematic data, whereas we tested the last hypothesis via analysis of survey responses.

## 2. Materials and Methods

### 2.1. Participant Recruitment

Based on the results of prior studies using similar experimental techniques [17,18], a convenience sample of 15 healthy right-handed individuals (8 female, 7 male; age range: 20–27 years) gave informed consent to participate in this study. All procedures were approved by the Institutional Review Board of Marquette University in compliance with the 1964 Declaration of Helsinki. None of the participants had a known history of neurological disease or sensory deficits, colorblindness, or reported visual difficulty. Each participant volunteered in two experimental sessions performed on separate days (day range: 2–17). Each session required about 1 to 1.5 h to complete, including experimental setup and testing.

### 2.2. Experimental Set-Up

**Figure 1:**
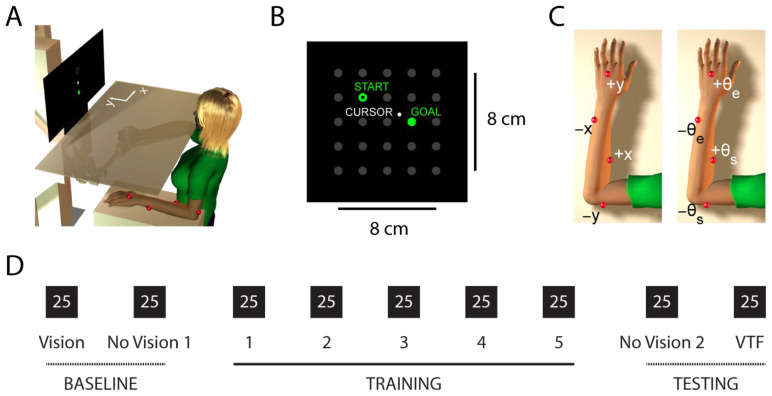
Experiment setup and protocol. (**A**) A participant grasping the handle of the planar manipulandum with the dominant right hand while seated in front of a grid of targets presented on a vertical computer display. An opaque horizontal shield obstructed their view of the physical workspace. Red markers indicate the location of eccentric rotating mass vibration motors (the vibrotactile display) fixed to the non-dominant arm. (**B**) Visual workspace showing the 5 × 5 grid of targets and a cursor corresponding to the position of the right hand in the physical workspace. (**C**) Location and information content of the individual vibration motors affixed to the non-preferred arm under the Cartesian Endpoint Encoding scheme (left) and Joint Angle Encoding scheme (right). (**D**) The structure of each of the two experimental sessions: Nine blocks of 25 reaches under 4 different feedback conditions.

We adapted techniques previously used to study the integration of supplemental vibrotactile kinesthetic feedback into the ongoing control of goal-directed actions [17,18]. Participants were seated in an adjustable, high-backed chair that was positioned directly in front of a horizontal planar robotic manipulandum (Figure 1A). They used their right hand to grasp a spherical handle affixed to the robot’s endpoint. Handle location was resolved within 0.038 mm using joint angular position data from two 17-bit encoders (A25SB17P180C06E1CN; Gurley Instruments Inc., Troy, NY, USA) [57]. Hand position data were collected at 200 samples/s using the MATLAB XPC real-time computing environment (The Mathworks Inc., Natick, MA, USA). An opaque, horizontal shield was placed directly above the plane of motion of the robotic arm to prevent a direct view of the participant’s arm and robot. The seat position and height were adjusted so that the participant was able to reach all positions of the workspace without moving the torso. Movement of the torso was further minimized using nylon shoulder restraints attached to the chair. The right arm rested in a four-degree-of-freedom, lightweight arm support that was fixed to the seat; this linkage acted to counteract the effect of gravity and to maintain a shoulder abduction angle of approximately 75° to 85°. Wrist motions were minimized using an orthopedic brace.

A vertical 40″ LED screen (Westinghouse WD40FX1170) was mounted approximately 70 cm directly in front of the participant and was used to present visual stimuli. Twenty-five visual targets (1 cm diameter) were arranged in a 5 × 5 grid that had an edge length of 8 cm (Figure 1B); we refer to this grid of targets as the visual workspace. During certain trials, hand position was represented onscreen with a white cursor (0.5 cm diameter) that provided honest real-time visual feedback of hand location relative to the visual targets. Hand motion in the physical workspace was mapped to cursor motion in the visual workspace in a 1:1 manner, whereby a 1 cm movement of the hand to the participant’s right corresponded to a 1 cm displacement of the cursor to the right. Likewise, a 1 cm movement of the hand away from the participant’s body corresponded to a 1 cm displacement of the cursor toward the top of the visual display.

We attached a “vibrotactile display” to the non-moving left arm and used it to provide supplemental kinesthetic feedback regarding the motion of the moving right hand (Figure 1A). The vibrotactile display was comprised of four eccentric rotating mass (ERM) vibration motors (Precision Microdrives Inc., London, UK; Model: 308–102), which have an operational frequency range of 60 Hz to 380 Hz and a covarying amplitude range of 0.0 to 6.3 G. Each vibration motor was encased in a polyolefin sheath that could be affixed directly to the skin of the arm with athletic tape and adhesive bandages. With reference to anatomic position, the placement of the four vibration motors, shown in Figure 1C, included the (i) C7 dermatome on the dorsal hand, about 5 cm proximal to the middle finger knuckle; (ii) C8 dermatome on the ventromedial forearm, approximately 5 cm proximal to the ulnar styloid process; (iii) C6 dermatome on the ventrolateral forearm, approximately 5 cm distal to the antecubital fossa; (iv) T1 dermatome on the posterior arm, about 5 cm proximal to the olecranon (cf., [58]). The spacing between motors was greater than 6 cm at all sites, thereby avoiding undesirable mechanical crosstalk between adjacent sites [59,60]. Participants wore noise-canceling headphones (Boltune, Cupertino, CA, USA; Model BT-BH011) that played white noise throughout experimental testing to minimize audible cues from the vibration motors and other potential audible distractions.

### 2.3. Vibrotactile Feedback Encoding Schemes

Instantaneous hand position, as measured by the horizontal planar robot, was encoded into supplemental vibrotactile feedback using two different encoding schemes: *Cartesian Endpoint Encoding* (CEE) and *Joint Angle Encoding* (JAE) (Figure 1C). In Cartesian Endpoint Encoding, the vibrotactile feedback was encoded in a Cartesian frame of reference with its origin in the center of the central target of the 5 × 5 grid, which we refer to as the “home position”. The motors did not produce any vibrations when the hand position was within 0.25 cm of the home position. Movement of the hand to the right of the home position produced vibrations in the X+ motor, whereas movement of the hand to the left of the home position produced vibrations in the X*−* motor (see Figure 1B,C). Likewise, hand movements that would move the cursor higher than the home position in the visual workspace corresponded to vibrations in the Y+ motor, and movements in the lower half of the workspace caused the Y*−* motor to vibrate. The X+ and X*−* motors were not activated with vertical hand movement along the Y-axis of the visual workspace (i.e., when X = 0). Similarly, the Y+ and Y*−* motors did not produce vibrations with horizontal hand movement along the X-axis in the visual workspace (i.e., when Y = 0). The vibrotactile feedback was encoded as a vector ranging from 75 Hz at 0.25 cm from the home position to 200 Hz at the center of the most distant target (4.0 cm from the home position). The vibrotactile feedback saturated at maximum activation at distances greater than 5.5 cm from the home position.

**Figure 2:**
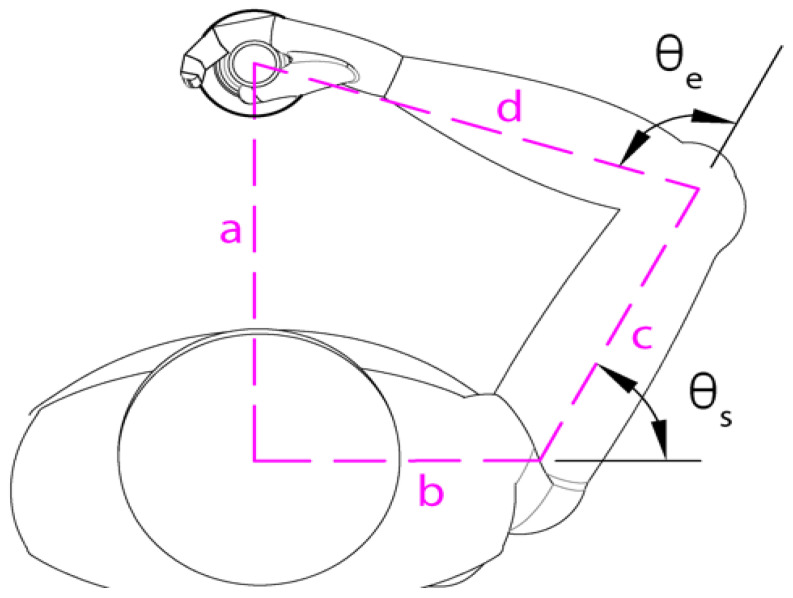
Measurements involved in the calculation of participant joint angles. With the participant grasping the handle at the home position, distance measurements were made between (a) the robot handle to the participant’s sagittal midline, (b) the participant’s sagittal midline to the shoulder’s center of rotation, (c) the shoulder’s center of rotation to the elbow’s center of rotation, and (d) the elbow’s center of rotation to the center of the robot handle. $\theta_{e}:$ elbow angle. $\theta_{s}:$ shoulder angle.

For Joint Angle Encoding, hand position as measured by the robot was used to infer the participant’s instantaneous shoulder angle (*q_s_*) and the elbow angle (*q_e_*) using inverse kinematics analysis [Equations (1a) and (1b)] and individualized measurements as described in Figure 2 (see also Appendix A). With the participant grasping the handle at the home position, measurements were taken between (a) the robot handle to the participant’s sagittal midline, (b) the participant’s sagittal midline to the shoulder’s center of rotation, (c) the shoulder’s center of rotation to the elbow’s center of rotation, and (d) the elbow’s center of rotation to the center of the robot handle. Measurements a and b were used to reframe the current hand position from the robot reference frame into a shoulder-centered reference frame (*x*,*y*) in real time. The distance (*h*) between the participant’s shoulder and hand was then calculated to find current shoulder and elbow joint angles using static measurements c and d.

(1a) $$\theta_{e}=tan^{-1}\left(\frac{y}{x}\right)-cos^{-1}\left(\frac{h^{2}+c^{2}-d^{2}}{2ch}\right)$$

(1b) $$\theta_{s}=\pi -cos^{-1}\left(\frac{h^{2}-c^{2}-d^{2}}{-2cd}\right)$$

Joint Angle Encoding utilized the same visual workspace as Cartesian Endpoint Encoding with comparable scaling such that vibrotactile feedback varied over the same range of intensities in the *q_e_−/+* or *q_s_−/+* motors in response to changes in the elbow or shoulder angles, respectively.

### 2.4. Testing Procedures

In one of the two sessions, we tested the capability of participants to use vibrotactile feedback with Cartesian Endpoint Encoding to enhance the accuracy and efficiency of goal-directed reaching movements. In the other session, we similarly tested vibrotactile feedback with Joint Angle Encoding. The order of the two sessions was counterbalanced across participants. Prior to testing on each day, participants were introduced to that day’s vibrotactile display, they were informed about how to interpret the vibrotactile cues, and they were invited to freely explore the robot’s workspace. During this familiarization period, participants were frequently and repeatedly asked to report which motors were activated at any given time. If they made errors in detecting vibration on any motor, the motor’s location was adjusted by 2 or 3 cm within the same dermatome so that each participant could reliably detect and report vibration. Participants were then encouraged to explore the vibrotactile display by making self-guided reaching movements until comfortable with the encoding scheme. This introduction and exploration procedure took between 2 to 5 min to complete and established how the workspace was encoded within the vibrotactile feedback.

During the main part of both experimental sessions, participants performed 9 blocks of 25 reach-to-target movements, one movement per trial (Figure 1D). Participants started each block with their right hand centered within the home position. The target grid was always displayed on-screen as a set of low-contrast gray dots, and the current target was presented in vivid green (Figure 1B). Participants were instructed to “capture the target as quickly and accurately as possible”. Upon completing a reach, they were to announce that they had arrived at the target, and the experimenter ended the trial unless 10 s had elapsed, whereupon the trial ended automatically. At the end of the trial, the previous target became an empty green dot, and after a random delay period (2.3 ± 0.7 s), a new location in the workspace turned green, cueing the participant to move to that location. Target sequences were pseudo-randomized across each block of 25 trials. The distance between consecutive targets ranged from 4.0 to 6.32 cm.

The block order and descriptions were as follows:

1. Visual Feedback: Participants were able to see a cursor representing their hand position in the visual workspace. No vibrotactile feedback was provided. This block served to familiarize participants with the reaching task. This was the only block where participants were able to see the cursor representing hand position in the visual workspace.

2. No Vision 1: Neither visual feedback nor vibrotactile feedback was provided. This block served to provide a baseline assessment of performance guided only by proprioceptive feedback (for comparison with blocks where visual or vibrational cues were also provided).

3–7. VTF Training 1–5: Participants completed 5 blocks of 25 reaches, each with supplemental vibrotactile feedback (VTF Training) and without concurrent visual feedback. If the center of the robot’s handle was more than 0.25 cm from the center of the target when the participants indicated they had completed a reach, the robot smoothly moved the hand to the center of the indicated target with a 1 s movement time. These training blocks were the only trials where terminal corrections were applied. No visual knowledge of results was provided during the training blocks in order to encourage participants to learn the mapping from hand position in the physical workspace to patterns of vibrotactile feedback within the vibrotactile display.

8. No Vision 2: Neither visual feedback nor vibrotactile feedback was provided. This block served to establish a post-training measure of performance guided only by proprioceptive feedback. No robot corrections were provided during this block to allow for a fair comparison with both the pre-training No Vision 1 block (to quantify the general benefits of training) and with the post-training VTF Test block (to quantify the benefits of concurrent vibrotactile guidance).

9. VTF Test: No visual feedback or robot corrections were provided during this final test of reach performance guided by concurrent vibrotactile feedback. We sought to compare performance improvements in this block relative to the No Vision 2 block across encoding schemes as a primary outcome of this study.

At the end of each session, participants completed three surveys that assessed their subjective experiences with each encoding scheme in terms of usability, motivation, and satisfaction. As described below, these included the System Usability Scale (SUS; [53]), the Intrinsic Motivation Inventory (IMI; [54]), and the Quebec User Evaluation of Satisfaction with Assistive Technology (QUEST 2.0; [55,56]).

### 2.5. Analysis of Kinematic Data

Analysis of kinematic performance focused principally on the *final hand position,* which was recorded when participants verbally indicated that they had acquired the intended target. Our primary measure of movement accuracy was *target capture error*, defined as the Euclidean distance between the center of the illuminated target and the final hand position. Our primary measure of movement efficiency was *target capture time*, defined as the time that elapsed from the moment the target was illuminated to when the participant gave a verbal cue to the researcher or after 10 s had expired, whichever came first. We also computed two secondary outcome measures pertaining to kinematic efficiency. One was the *normalized path length*, defined as the total hand path length within a trial divided by the length of the ideal straight-line path between the hand’s starting location and the desired target. Another was a *decomposition index* [DI; Equation (2)] that is sensitive to different strategies for using vibrotactile feedback to solve the target capture task in the absence of visual feedback [18]. The decomposition index is a unitless measure that quantifies the extent to which sampled-data hand paths in any given trial move exclusively parallel to the cardinal axes of the vibrotactile display. In the case of Cartesian Endpoint Encoding, for example, the cardinal axes of the vibrotactile display correspond to the {*x*, *y*} axes of the visual display. If we let the generalized variable *q*_1_ correspond to the hand’s *x*-axis location and the variable *q*_2_ correspond to its *y*-axis location, we obtain:

(2) $$DI=\overset{N}{\underset{n=2}{\sum }}\;\left\{\frac{1}{2}\left|\frac{q_{1}\left(n\right)-q_{1}\left(n-1\right)}{\sum_{n=2}^{N}\left\{q_{1}\left(n\right)-q_{1}\left(n-1\right)\right\}}\right|\left|\frac{{\dot{q_{2}}}_{max}-\dot{q_{2}}\left(n\right)}{{\dot{q_{2}}}_{max}}\right|+\frac{1}{2}\left|\frac{q_{2}\left(n\right)-q_{2}\left(n-1\right)}{\sum_{n=2}^{N}\left\{q_{2}\left(n\right)-q_{2}\left(n-1\right)\right\}}\right|\left|\frac{{\dot{q_{1}}}_{max}-\dot{q_{1}}\left(n\right)}{{\dot{q_{1}}}_{max}}\right|\right\}$$

where *N* corresponds to the maximum number of data samples within a given trajectory, n is the sample number within that trajectory, and ${\dot{q_{1}}}_{max}$, ${\dot{q_{2}}}_{max}$ correspond to the peak hand speeds along the cardinal {*x*, *y*} axes. For computing the decomposition analysis in trials with Joint Angle Encoding, the variable *q*_1_ corresponds to the shoulder angle *q_s_*, and *q*_2_ corresponds to the elbow angle *q_e_*. Hand movements represented as smooth straight lines in joint angle coordinates (e.g., [61]) have low decomposition index values, whereas movements comprised of sequential motions at the two joints have high decomposition index values. Per Equation (2), off-axis, straight-line trajectories with bell-shaped (Gaussian) velocity profiles in the {*q*_1_, *q*_2_} reference frames yield decomposition index values equal to 0.19, whereas there is no upper limit on decomposition index values for highly decomposed movements.

### 2.6. Assessment of Subjective User Experience

Usability is defined as the “appropriateness to a purpose of any particular artefact” [53]. We used the System Usability Scale (SUS; [53]) to assess the usability of each vibrotactile feedback encoding scheme within the context of enhancing the accuracy and efficiency of goal-directed reaching movements. The SUS is a 10-item questionnaire using a 5-option Likert scale (ranging from “Strongly Disagree” to “Strongly Agree”). Scores were summed across items and scaled to yield a total score ranging from 0 to 100. Higher scores indicate better usability, with scores greater than 68 generally regarded as indicating passable usability [62].

We used a 30-question subset of the Intrinsic Motivation Inventory (IMI; [54]) to assess the extent to which participants perceived the supplemental kinesthetic feedback to be motivating. The questionnaire spanned five dimensions of the original survey, including “interest/enjoyment”, “effort/importance”, “value/usefulness”, “perceived competence”, and “felt pressure/tension.” The remaining two sections of the original survey, “perceived choice” and “relatedness”, were removed because they assessed aspects of user interaction beyond the scope of this study. Participants responded to each of the 30 prompts using a 7-option Likert scale (ranging from “Not At All True” to “Very True”). The IMI “interest/enjoyment” subscale is generally considered to be the self-report measure of intrinsic motivation [63,64]. Higher scores on most subsections indicate better subjective user experience (except for the “pressure/tension” subsection, where higher scores indicate that the participant felt more pressured or tense during the exercise). Average “interest/enjoyment” scores greater than or equal to four indicate that a given system is perceived as being motivated to use.

Finally, we assessed user satisfaction using the Quebec User Evaluation of Satisfaction with Assistive Technology (QUEST 2.0; [55,56]). We used an 8-item version of the QUEST that assessed user satisfaction in terms of the system’s physical characteristics (e.g., size, weight), physical and cognitive fit (e.g., physical comfort, ease in adjusting, ease in learning), and functional characteristics (e.g., how successfully the device performed); we did not include the original QUEST subsection that assesses satisfaction with services associated with the device because such services were neither rendered nor necessary for the completion of our study. The QUEST questionnaire used a 5-option Likert scale ranging from “Not Satisfied At All” to “Very Satisfied”. Higher scores indicate higher user satisfaction. The individual item scores were averaged across the eight questions to obtain a final score. Scores equal to or greater than 3 were considered to indicate passable satisfaction with the assistive device. Lastly, the QUEST asked participants to identify from a list of eight options (12 in the original questionnaire; 4 pertaining to services were removed) the three most important aspects of a wearable vibrotactile feedback system that would impact their satisfaction in using it.

### 2.7. Statistical Hypothesis Testing

This study tested three main hypotheses. The first pose is that after a brief training period (~30 min), supplemental vibrotactile feedback can improve reach accuracy in the absence of concurrent visual feedback for both encoding schemes. Given the within-subject research design, this hypothesis was tested using planned one-sided paired *t*-tests to compare target capture error across the No Vision 2 and VTF Test blocks within each encoding scheme. The second hypothesis poses that when reaching without vision, one or the other vibrotactile encoding schemes will promote better reaching accuracy and/or efficiency. On the one hand, intrinsic proprioceptors normally used for real-time control of arm movements are embedded in muscles spanning the shoulder and elbow joints, suggesting that supplemental vibrotactile feedback encoded in a joint angle coordinate system may be more intuitive and effective to use. On the other hand, Cartesian Endpoint Encoding of supplemental vibrotactile feedback largely conforms to the visual reference frame, and so vibrotactile feedback encoded in Cartesian coordinates could be more effective and easier to use due to vision’s dominant influence on the specification of movement vectors [1]. To test this hypothesis, we performed planned, paired-sample *t*-tests comparing the primary performance measures across the vibrotactile feedback encoding schemes during the VTF Test blocks. The third hypothesis predicts that participants will prefer one way of encoding supplemental vibrotactile feedback over the other (i.e., that user experience will differ between the Cartesian Endpoint and Joint Angle Encoding schemes). To test this hypothesis, we operationally defined a “more positive user experience” as one where participants would find the system to be more usable, more motivating, and/or more satisfying. Separate planned paired-samples *t*-tests were therefore performed to determine whether SUS values, IMI values, and QUEST values systematically favored Joint Angle Encoding or Cartesian Endpoint Encoding. Subsequently, 95% confidence intervals (CI) were calculated to classify cohort subjective experiences. Secondary outcome measures were analyzed using repeated measures ANOVA and post-hoc paired-samples *t*-tests to compare performance across trial blocks and encoding schemes. Statistical testing was performed in R Studio 2022.07.1. Statistical significance was set at a family-wise error rate of α = 0.05.

## 3. Results

All participants completed both testing sessions (Cartesian Endpoint Encoding and Joint Angle Encoding), including post-test surveys, and all were attentive throughout the study. As such, data from all participants were included in the statistical hypothesis testing described in the following paragraphs.

### 3.1. Effects of Supplemental Vibrotactile Feedback on Primary Measures of Reach Accuracy and Efficiency

**Figure 3:**
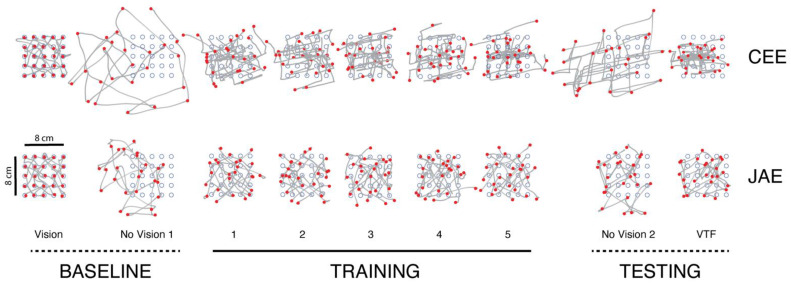
Representative hand paths (grey) and final positions (red) in all trial blocks performed by selected participants in the two encoding schemes. (**Top**): Cartesian Endpoint Encoding (CEE); (**Bottom**): Joint Angle Encoding (JAE). Each block is shown as a separate plot with block conditions labeled below. Block conditions with dashed delineators (i.e., baseline, testing) were performed without robotic repositioning of the hand at the end of each reach. Blocks with the solid delineator above the label (training) were subject to the robotic repositioning of the hand at the end of each reach. Five training blocks were performed. Horizontal and vertical scale bars: 8 cm.

Figure 3 shows examples of hand paths (gray traces) and movement endpoints (red dots) relative to the workspace targets (blue circles) within all nine trial blocks performed by selected participants for each of the two experimental sessions: Cartesian Endpoint Encoding and Joint Angle Encoding. During baseline testing with concurrent visual cursor feedback (Vision), the hand and its cursor moved directly from one target to the next, following approximately straight paths. All movement endpoints were within their intended targets. Upon removing the cursor (No Vision 1; in the absence of supplemental vibrotactile feedback), hand paths failed to achieve their targets. Instead, movements became much longer than ideal such that target capture errors accumulated from one movement to the next. In both sessions, the space spanned by the hand paths drifted to the left (i.e., toward the participant’s midline) and greatly exceeded the space spanned by the target set. These features of disordered reaching decreased immediately upon providing supplemental vibrotactile feedback in both sessions. Recall that the robot repositioned the hand to the intended target at the end of each reach in the training blocks; this provided proprioceptive knowledge of results that participants could use to learn how hand location in the physical workspace relates to patterns of vibrations in the vibrotactile display. Passive repositioning of the hand also minimized the accumulation of errors from one trial to the next within each training block, i.e., proprioceptive drift [65]. Interestingly, VTF Training trials performed with a Cartesian Endpoint Encoding of hand position appear to be decomposed into separate horizontal (*x*-axis) and vertical (*y*-axis) motions. Although less apparent from these hand path plots, the participant also tended to decompose training reaches into separate motions at the shoulder and elbow joints in the Joint Angle Encoding session. With Cartesian Endpoint Encoding, decomposition appeared to persist after training was completed, even when vibrotactile feedback was removed in the No Vision 2 block. However, this effect was not significant for Joint Angle Encoding. For both encoding schemes, movement accuracy degraded in the absence of vibrotactile feedback and concurrent vision in the No Vision 2 Test block. By contrast, movement accuracy improved considerably when vibrotactile feedback was re-instated in the VTF Test block, even without robotic repositioning of the hand at the end of each reach, which was limited to the five training blocks.

**Figure 4:**
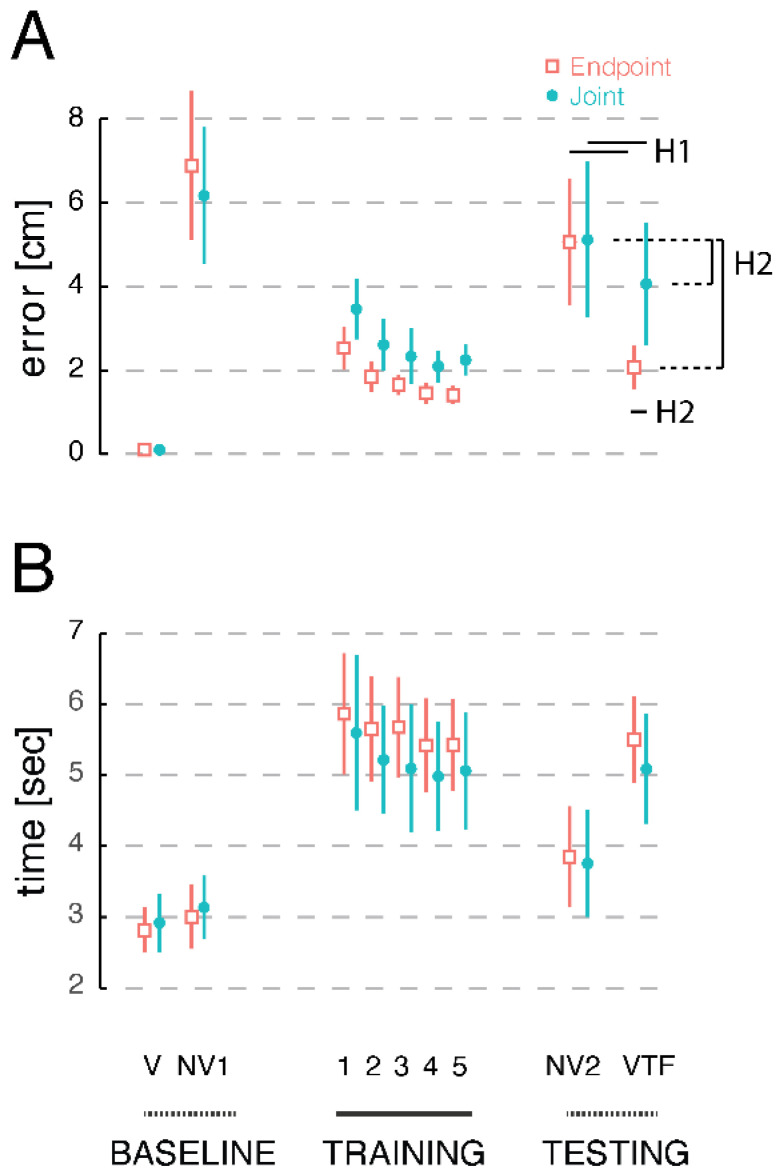
Cohort results: primary outcome measures for each trial block in each experimental session. (**A**) target capture error; (**B**) target capture time. Orange open squares: average performance in the Cartesian Endpoint Encoding session (CEE). Teal solid circles: average performance in the Joint Angle Encoding session (JAE). The training blocks, marked with a solid black line on the *x*-axis, included robotic repositioning of the hand to the intended target at the end of each trial. Baseline and testing trials did not include robotic repositioning. Error bars: 95% CI of the mean.

The single-subject trends depicted in Figure 3 were characteristic of movements made by the entire study cohort. Figure 4A presents the across-participants average target capture error (our primary outcome measure of reach accuracy) for each trial block in both sessions. Target capture errors were minimal (averaging only 0.1 ± 0.03 cm, mean ± SD) when participants could view an onscreen cursor that represented the location of their moving hand in the workspace. Reach accuracy degraded markedly when the cursor was removed in the No Vision 1 trial block (CEE: 6.9 ± 3.2 cm; JAE: 6.2 ± 2.9 cm). Accuracy improved substantially during initial training with vibrotactile feedback (Training Block 1), regardless of the encoding scheme (CEE: 2.5 ± 0.9 cm; JAE: 3.5 ± 1.3 cm). Accuracy improved progressively throughout the five training blocks, although the magnitude of target capture errors in the Joint Angle Encoding session appeared to exceed those in the Cartesian Endpoint Encoding session at the end of the training blocks (CEE: 1.4 ± 0.4 cm; JAE: 2.2 ± 0.7 cm). When vibrotactile feedback was removed in the No Vision 2 Test block, target capture errors increased dramatically in both sessions (CEE: 5.1 ± 2.7 cm; JAE: 5.1 ± 3.4 cm). Whereas average target capture errors decreased upon reinstating vibrotactile guidance in both sessions, there was a greater decrease with Cartesian Endpoint Encoding than Joint Angle Encoding (CEE: 3.1 ± 1.0 cm; JAE: 4.1 ± 2.6 cm).

We used a planned, one-sided, paired-sample *t*-test to test our first hypothesis (i.e., after a brief training period, both forms of supplemental vibrotactile feedback facilitate reach accuracy in the absence of concurrent visual feedback). To do so, we compared target capture errors across the No Vision 2 and VTF Test blocks for each participant and for each experimental session. Consistent with the hypothesis, both encoding schemes were effective at reducing target capture error in the absence of visual feedback (CEE: t_14_ = 4.56, *p* < 0.001; JAE: t_14_ = 2.34, *p* = 0.017), even in the absence of passive robotic repositioning of the hand, which was present during the VTF Training blocks but absent in the VTF Test block.

Figure 4B presents the across-participant’s average target capture times (our primary outcome measure of temporal efficiency) for each trial block in both experimental sessions. Target capture times were uniformly low when participants performed baseline reaches with and without concurrent cursor feedback of hand position (Vision—CEE: 2.8 ± 0.6 s; JAE: 2.9 ± 0.7 s. No Vision 1—CEE: 3.0 ± 0.8 s; JAE: 3.1 ± 0.8 s). By contrast, target capture times exceeded 5 s in both sessions, even at the end of training (CEE: 5.4 ± 1.2 s; JAE: 5.1 ± 1.5 s). Although capture times dropped nearly to baseline levels in the No Vision 2 block (CEE: 3.9 ± 1.3 s; JAE: 3.8 ± 1.4 s), they increased to end-of-training levels in the VTF Test block (CEE: 5.5 ± 1.1 s; JAE: 5.1 ± 1.4 s). This decrease in temporal efficiency suggests that the integration of supplemental vibrotactile feedback into the ongoing control of movement comes at a significant cost in terms of cognitive workload.

We used a set of planned, two-sided, paired-sample *t*-test to assess our second hypothesis (i.e., when reaching without vision, one or the other vibrotactile feedback encoding schemes would promote better reaching accuracy and/or efficiency). Here, we compared our primary measures of reach accuracy (target capture error) and efficiency (target capture time) across the two vibrotactile feedback encoding schemes in the VTF Test blocks. We found that Cartesian Endpoint Encoding was better than Joint Angle Encoding in enabling participants to reduce target capture errors, not only in absolute terms (i.e., comparing VTF Test blocks: t_14_ = 3.33, *p* < 0.005) but also in terms of error reduction relative to the No Vision 2 test block in each session (t_14_ = 3.81, *p* < 0.002). A similar trend toward better temporal efficiency with Cartesian Endpoint Encoding did not quite achieve statistical significance (comparing VTF Test blocks: t_14_ = 1.89, *p* = 0.080). It should also be noted that VTF Test block capture times were considerably longer than capture times in the No Vision 2 test blocks for both the Cartesian Endpoint (t_14_ = 7.93, *p* < 0.001) and Joint Angle (t_14_ = 3.92, *p* < 0.002) sessions.

### 3.2. Secondary Analyses of Kinematic Performance during Reaching with Supplemental Vibrotactile Feedback

**Figure 5:**
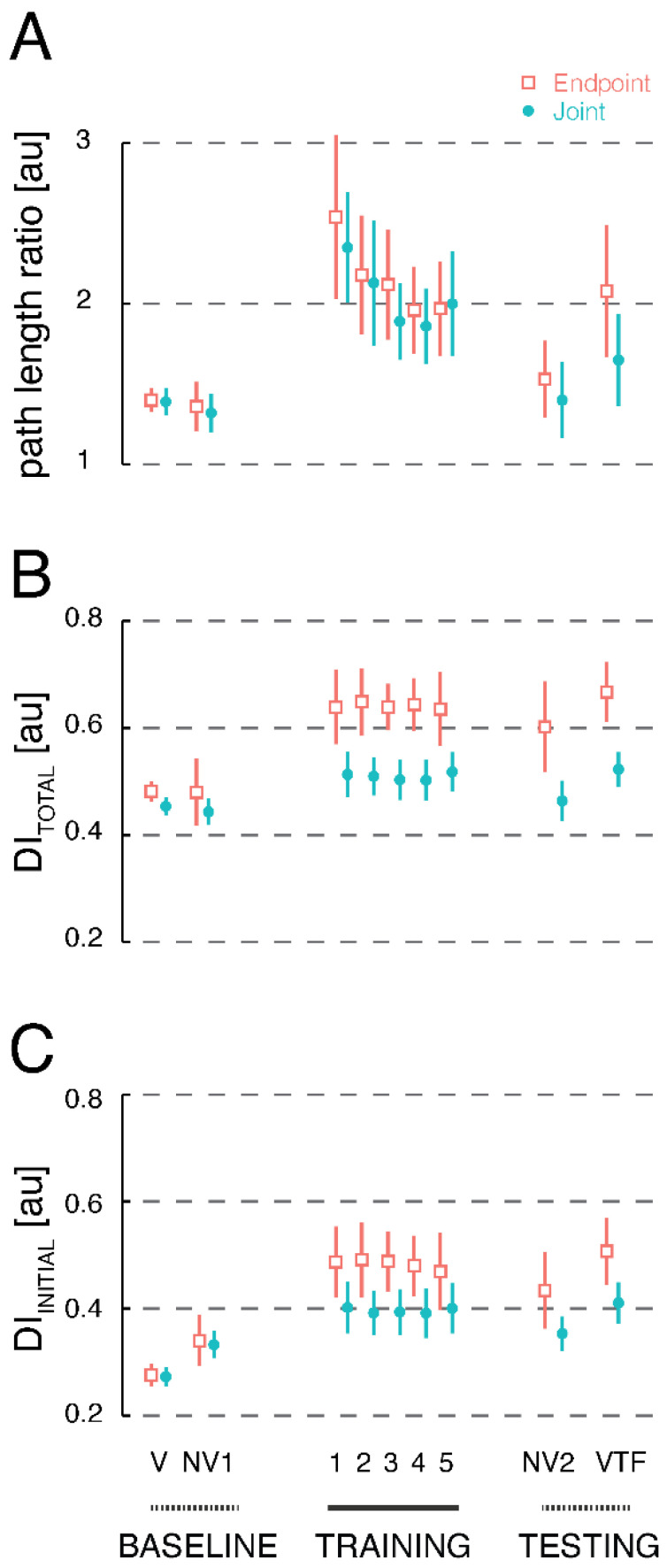
Cohort results: secondary measures of spatial efficiency. Panel (**A**) (top) shows the cohort average path length ratio averaged across trials within each block; the cohort mean (symbols) and 95% CI of the cohort mean (error bars) for all blocks of endpoint encoding (orange) and joint encoding (teal). Panel (**B**) (middle) shows the cohort average decomposition index for the entire distance traveled in each trial with 95% CI of the mean for all blocks of endpoint and joint encoding. Panel (**C**) (bottom) shows the cohort average decomposition index for the initial half of the distance traveled in each trial for each block in endpoint and joint encoding. The training blocks, marked with a solid black line along the *x*-axis, included robot corrections to target at the end of trials.

We analyzed secondary measures of kinematic performance (path length ratio and decomposition index) to gain insight into strategies participants may have used to integrate supplemental vibrotactile kinesthetic feedback into the ongoing control of reaching. Figure 5A presents the cohort-average path length ratio, a measure of the spatial efficiency of reaching, for each trial block in both experimental sessions. Average path length ratios were uniformly low (nearly ideal) when participants could view a cursor representing the location of their hand within the workspace (CEE: 1.4 ± 0.13; JAE: 1.39 ± 0.15). The spatial efficiency of reaching was preserved when the cursor was removed in the No Vision 1 trial block (CEE: 1.4 ± 0.28; JAE: 1.3 ± 0.22). By contrast, path length ratios increased markedly during initial training with vibrotactile feedback (Training Block 1), regardless of the encoding scheme (CEE: 2.5 ± 0.92; JAE: 2.4 ± 0.62). The efficiency of reaching increased progressively throughout the five training blocks, with path length ratios achieving nearly identical values in the two sessions at the end of training (CEE: 2.0 ± 0.59; JAE: 2.0 ± 0.53). When vibrotactile feedback was removed in the No Vision 2 test block, path length ratios dropped to near baseline levels (CEE: 1.5 ± 0.43; JAE: 1.4 ± 0.43). Although the average path length ratio appeared to increase upon reinstating vibrotactile guidance with Cartesian Endpoint Encoding (CEE: 2.1 ± 0.74), Joint Angle Encoding did not tend to demonstrate as large an increase (JAE: 1.7 ± 0.52 cm).

Because we did not have specific hypotheses pertaining to the secondary performance measures, we performed an omnibus repeated measures, 2-way ANOVA that examined the impact of the block number and encoding scheme on path length ratio; we found significant effects of both factors (Trial Block: F_(8,238)_ = 29.19, *p* < 0.0005; Encoding Scheme: F_(1,238)_ = 8.32, *p* = 0.004), but no interaction between them (F_(8,238)_ = 0.95, *p* = 0.48). Notably, path length ratios decreased significantly from the start to end of training for both encoding schemes (CEE: t_14_ = 3.78, *p* = 0.002; JAE: t_14_ = 4.07, *p* = 0.002). After training, VTF Test block reaches with Joint Angle Encoding exhibited lower path length ratios than did reaches with Cartesian Endpoint Encoding (t_14_ = 3.16, *p* = 0.007), although neither form of vibrotactile guidance promoted test block reaches with path length ratios as low as those observed in the No Vision 2 trial blocks (CEE: t_14_ = 2.89, *p* = 0.012; JAE: t_14_ = 4.19, *p* < 0.001).

We used the decomposition index of Equation (2) to quantify the extent to which vibrotactile-guided reaching tended to be decomposed into separate motions along the cardinal axes of the vibrotactile display. Figure 5B presents average decomposition index values for each trial block as computed from the moment the target was illuminated to when the participant’s hand speed dropped below 10% of its peak value on that trial (i.e., the total movement). In both sessions, DI _Total_ values were uniformly low when participants could view a cursor representing the location of their hand within the workspace (Vision block: CEE: 0.48 ± 0.03; JAE: 0.45 ± 0.03) and when the cursor was removed in the No Vision 1 trial block (CEE: 0.48 ± 0.11; JAE: 0.44 ± 0.04). By contrast, DI _Total_ values increased at the onset of training (Training block 1: CEE: 0.64 ± 0.12; JAE: 0.51 ± 0.08) and remained elevated through the end of training (Training block 5: CEE: 0.64 ± 0.12; JAE: 0.52 ± 0.07). Relative to the No Vision 1 trial block, the extent to which DI _Total_ values increased during vibrotactile-guided reaching appeared to be greater for Cartesian Endpoint Encoding than Joint Angle Encoding. After training with Cartesian Endpoint Encoding, DI _Total_ values remained elevated both in the No Vision 2 block (CEE: 0.60 ± 0.15) and in the VTF Testing block (CEE: 0.67 ± 0.10). By contrast, elevated decomposition index values did not carry over into the No Vision 2 block after training with Joint Angle Encoding (JAE: 0.46 ± 0.07) but instead re-emerged in the VTF Testing block (JAE: 0.52 ± 0.06). These observations were supported by results of repeated measures, 2-way ANOVA that examined the impact of block number and encoding scheme on DI _Total_ values; we found significant effects of both factors (Trial Block: F_(8,238)_ = 38.06, *p* < 0.001); Encoding Scheme: F_(1,238)_ = 106.77, *p* < 0.001), as well as an interaction between the two factors (F_(8,238)_ = 3.47, *p* < 0.001). We noted no significant improvement in the decomposition index from the first to the fifth VTF Training block for either Cartesian Endpoint Encoding (t_14_ = 0.19, *p* = 0.85) or Joint Angle Encoding (t_14_ = 0.50, *p* = 0.63). After training, reaching movements remained significantly more decomposed in the No Vision 2 block relative to the No Vision 1 block for Cartesian Endpoint Encoding (t_14_ = 3.35, *p* < 0.005) but not Joint Angle Encoding (t_14_ = 1.68, *p* = 0.115). VTF Test blocks had significantly higher decomposition index compared to respective No Vision 2 blocks for both encoding schemes (CEE: t_14_ = 2.99, *p* < 0.01; JAE: t_14_ = 4.23, *p* < 0.001). DI _Total_ values were significantly higher for Cartesian Endpoint Encoding than for Joint Angle Encoding in the VTF Test blocks (t_14_ = 7.4, *p* < 0.001).

To address the possibility that DI _Total_ values might be dominated by iterative feedback corrections that were sometimes observed at the end of the reaching movements, we repeated the decomposition index analysis using hand path data from only the first half of each trial (i.e., DI _Initial_; Figure 5C). Although not shown here, the statistical analysis yielded a similar pattern of results as described in the previous paragraph, suggesting that participants used a decomposition strategy from the start of movement under both encoding schemes, although the kinematic effects of this strategy were strongest with Cartesian Endpoint Encoding.

### 3.3. Subjective User Experience between Cartesian Endpoint Encoding and Joint Angle Encoding

**Figure 6:**
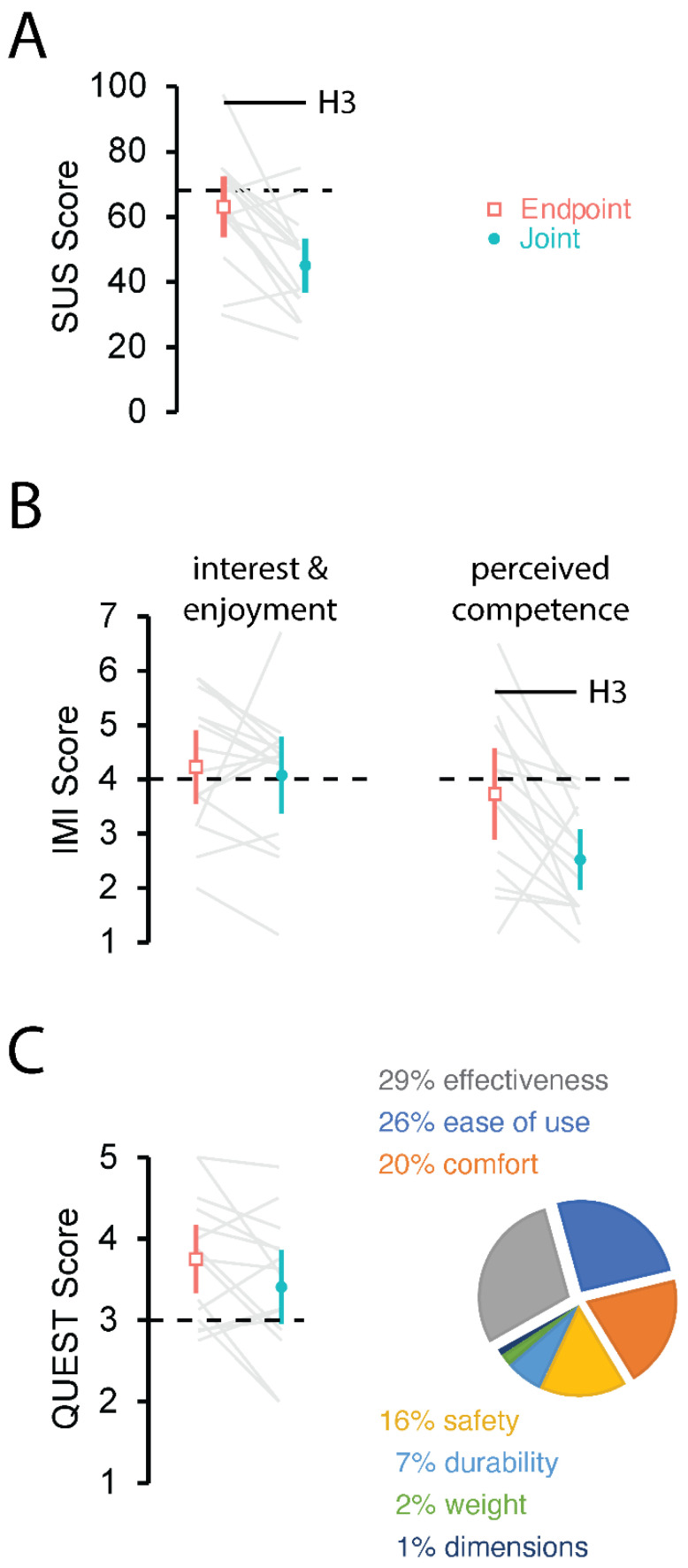
Subjective user experience. Survey results for SUS (**A**), IMI (**B**) and QUEST (**C**) show average cohort scores with 95% CI of the mean for the endpoint (orange open square) and joint (solid teal circle) encoding. Solid gray lines link the corresponding scores of each encoding method from individual participants. Dashed lines represent the scores that demarcate positive experience thresholds. The pie chart shows the most important attributes of an assistive device as indicated by participants on the QUEST. The pie chart values shown here were rounded to the nearest integer percentage.

We next assessed subjective user experiences after one day of exposure to each form of information encoding (Figure 6). We used the System Usability Scale (SUS) as our primary measure of system usability. Although there was considerable variation in SUS scores across the study cohort, participants generally rated Cartesian Endpoint Encoding as more usable than Joint Angle Encoding (Figure 6A: CEE: 63 ± 17; JAE: 45 ± 15; t_14_ = 4.3, *p* = 0.001). Of the two encoding schemes, only Cartesian Endpoint Encoding achieved a mean SUS score that included the threshold of passable usability.

We used the Intrinsic Motivation Inventory (IMI) as our primary measure of the system’s ability to motivate use. Of the five IMI subscales assessed at the end of each session (see Table 1), only the “perceived competence” subscale exhibited a significant difference between the two encoding methods (t_14_ = 3.3, *p* = 0.005; Figure 6B right); participants felt significantly more competent using Cartesian Endpoint Encoding compared to Joint Angle Encoding. The 95% CI for Cartesian Endpoint Encoding included the threshold of satisfactory perceived competence, whereas the 95% CI for Joint Angle Encoding did not. By contrast, we found no compelling evidence of significant differences between encoding methods for any of the remaining IMI subsets (t_14_ < 1.92, *p* > 0.07 in all four cases). In particular, we note that for both encoding schemes, average scores for the “interest/enjoyment” subscale exceeded the threshold value demarcating whether or not the task was motivating (Figure 6B left).

We used the Quebec User Evaluation of Satisfaction with Assistive Technology (QUEST) to assess each user’s perceived satisfaction with each of the two movement encoding schemes. Eleven of the 15 participants indicated that they found Cartesian Endpoint Encoding to be more satisfactory than Joint Angle Encoding (Figure 6C, left). On average, QUEST scores equaled or exceeded the threshold value of 3.0, indicating acceptable user satisfaction for both encoding schemes (CEE: 3.8 ± 0; JAE: 3.4 ± 0.8). The difference in satisfaction between the two encoding methods did not reach significance (t_14_ = 2.0, *p* = 0.066). Finally, QUEST identified effectiveness, ease of use, and physical comfort as the three most important aspects of the system impacting participant satisfaction in using it.

In summary, only Cartesian Endpoint Encoding provided a satisfactory user experience in the sense that participants found Cartesian Endpoint Encoding but not Joint Angle Encoding to have passable usability. Moreover, while both encoding schemes yielded IMI scores suggesting that they were marginally motivating, participants felt more competent using Cartesian Endpoint Encoding as compared to Joint Angle Encoding. Both encoding schemes appeared to generate acceptable user satisfaction, as determined by scores on the QUEST survey.

## 4. Discussion

We compared the objective utility and subjective user experience of two different forms of supplemental kinesthetic feedback with regard to their ability to enhance the accuracy and efficiency of goal-directed reaching in the absence of visual feedback in healthy, neurologically intact adults. One form of feedback, Cartesian Endpoint Encoding, converted real-time hand position in a Cartesian frame of reference into supplemental kinesthetic feedback provided by a vibrotactile display attached to the non-moving arm and hand. The other encoding approach, Joint Angle Encoding, provided real-time arm configuration information via the vibrotactile display.

After a brief training period, both forms of supplemental feedback promoted improved reach accuracy in the absence of concurrent visual feedback over levels of performance achieved using proprioception alone. We found that both encoding schemes had merit, depending on which performance metric was examined. On the one hand, Cartesian Endpoint Encoding was better than Joint Angle Encoding in enabling participants to reduce target capture errors, not only in absolute terms but also in terms of error reduction relative to a control condition wherein neither visual feedback nor vibrotactile feedback was provided. However, accuracy improvements came at a cost in terms of temporal efficiency. Target capture times during vibrotactile-guided reaching were considerably longer than capture times in the No Vision Test Blocks for both encoding schemes. Neither encoding scheme yielded movements that were particularly smooth; in both cases, vibrotactile-guided reaches tended to be decomposed into distinct movements along the principal axes of the vibrotactile display, although movements made with Joint Angle Encoding did so to a lesser degree than movements with Cartesian Endpoint Encoding. These efficiency results suggest that integration of supplemental vibrotactile feedback into ongoing control of movement is cognitively demanding, at least within the time frame of this study (i.e., one brief training session with each form of feedback). Finally, we analyzed participant responses to standard surveys to assess subjective experience in terms of usability, motivation, and user satisfaction. Although participant responses suggest that both encoding schemes were motivating and that both yielded passable user satisfaction scores, only Cartesian Endpoint Encoding was found to have passable usability; participants felt more competent using Cartesian Endpoint Encoding than Joint Angle Encoding. Although a case could be made that Joint Angle Encoding promoted smoother movements, future projects aiming to develop wearable technology to enhance the terminal accuracy of goal-directed reaching may wish to focus on Cartesian Endpoint Encoding for continuous supplemental kinesthetic feedback, especially considering the user perceptions of effectiveness, ease of use, and comfort reported here.

### 4.1. Information Encodings for Supplemental Guidance of Movement

The idea of providing meaningful supplemental feedback to mitigate sensory loss (sensory substitution) or impairment (sensory augmentation) has been pursued for decades (see [36] for a review). While early investigations were largely confined to the research laboratory [66,67,68], advances in mobile computing systems have made it possible to create a variety of novel wearable augmentation technologies, including those intended for use by the visually impaired [69,70], patients with balance deficits [15,16,71], or users of myoelectric forearm prostheses [72]. The information provided as feedback should also be intuitive to interpret (cf., [73,74]) in the sense that the user should be able to detect and/or discriminate stimuli of practical relevance [75]. Of the many ways to deliver supplemental feedback, we proposed that vibrotactile stimulation poses the least risk for skin breakdown or interference with other senses and activities of daily living. Consequently, we focus the remainder of our discussion on issues pertaining to the provision of informative vibrotactile feedback. We do not consider here the application of vibrotactile stimuli absent of meaningful information encodings (e.g., stochastic resonance), which has been described in detail elsewhere (cf., [34,76,77]).

The literature describes several ways to encode relevant information into vibrotactile feedback for the guidance of movement. One characteristic that is useful for classifying encoding schemes pertains to whether the feedback is intended to be provided periodically or continuously. Periodic feedback has been used to convey symbolic information representing complex concepts or messages [78] or to alert users to an important event or sudden change in task conditions [48,79]. In one example, Cuppone and colleagues describe a system that can promote proprioceptive learning of wrist movements in the absence of vision [12,80,81]. After three to five days of wrist movement training wherein participants were given vibrotactile cues when wrist trajectory errors exceeded certain limits, neurologically intact adults demonstrated improvements in wrist proprioceptive acuity that were retained up to 10 days after training ceased [81]. By contrast, continuous feedback systems are designed to enhance sensorimotor control by providing continuous feedback on some aspects of behavior. Exemplar applications include postural stabilization in patients with vestibular deficits [15,16,71], grasp force regulation and hand aperture control in users of myoelectric forearm prostheses [72], waypoint navigation [22], and the training of specific patterns of limb movements [13,14,17,32,47,82]. Because a long-term goal of our work is to re-establish real-time feedback control of limb movements in patients with neuromotor injury via supplemental kinesthetic feedback, we limit further discussion to issues relevant to continuously wearable sensory substitution/augmentation technologies.

Two main types of information encoding schemes are used for continuous supplemental feedback. These include state-based approaches, which inform the user about a limb’s current position in space relative to some fixed reference configuration (e.g., [18]), and “goal-aware” approaches that either inform the user about the difference (error) between current and desired body configurations (cf., [14,16]) or about how the user should ideally move their body to achieve some task goal (cf., [83]). Krueger and colleagues recently compared the ability of neurologically healthy people to use limb state information or hand position error to enhance the performance of stabilization and reaching tasks performed with the arm [17]. The authors compared objective performance using measures of kinematic error, and they compared subjective assessments of usefulness provided on a 7-point Likert scale. Both encoding schemes were found capable of enhancing stabilization and reaching performance in the absence of vision, although error encoding yielded somewhat superior outcomes-objective and subjective due to the additional task-relevant information it contains [17]. However, the state feedback approach is the simpler of the two from an implementation perspective. Whereas it is relatively straightforward to estimate limb orientation and hand position using low-cost wearable inertial measurement systems (see [37] for a relevant review), it is a much harder problem to infer with accuracy the user’s intended movement objective at any given time. For the near future, at least, limb state encoding will have greater practical utility for wearable supplemental feedback systems.

The current study compared two approaches regarding how limb states could be best encoded. On the one hand, Joint Angle Encoding imitates the way limb state information is provided by intrinsic muscle spindle proprioceptors, which provide feedback about joint angles in the limb via signals sensitive to muscle stretch. Joint Angle Encoding is also easy to implement using wearable IMUs, which can provide real-time estimates of limb segment angles relative to a user-defined reference configuration. On the other hand, Cartesian Endpoint Encoding mimics how the cursor tracks changes in hand position via movement on the visual display. The VTF Testing results depicted in Figure 4A strongly favor Cartesian Endpoint Encoding if target capture accuracy is a priority. By contrast, the VTF Testing results depicted in Figure 5 favor Joint Angle Encoding if spatial efficiency of movement is a priority. The use of either encoding scheme incurred a steep cost in terms of movement time (Figure 4B; see also [84]), although this aspect of performance may be expected to improve with additional practice. In one recent study, participants trained for approximately 10 h on using Cartesian Endpoint Encoded supplemental kinesthetic vibrotactile feedback to guide goal-directed reaching in the horizontal plane [19]. As we also showed in the current study, the initial performance of vibrotactile-guided reaching demonstrated that people were able to rapidly interpret and use supplemental vibrotactile feedback to improve reaching accuracy, although in doing so, movement durations effectively doubled. Throughout the course of extended training, however, Shah and colleagues found that movement durations for vibrotactile-guided reaches asymptotically approached those for movements made in baseline conditions performed without either vibrotactile or visual feedback [19]. Although no long-term training study has yet been performed using supplemental kinesthetic vibrotactile feedback with Joint Angle Encoding, we expect that a similar pattern of improvement will evolve as experience accrues.

While several previous studies have surveyed user perspectives on sensory augmentation technologies and approaches, most utilized either a single standardized questionnaire or in-house, open-ended questions to determine subjective user experience [85,86,87]; in contrast, the current study used three formal and reliable surveys (SUS, IMI, QUEST) to quantify the usability, intrinsic motivation, and satisfaction of each vibrotactile feedback encoding method. Just as patients are encouraged to assume an active role in their healthcare, it is imperative to consider the user’s perspective when designing assistive devices. Satisfaction is one of the most important indicators of quality healthcare [88], and patients who are more satisfied may be more likely to utilize healthcare systems [89]. Additionally, satisfaction, along with effectiveness and efficiency, are key factors that influence the usability of a system [53]. Intrinsic motivation in the context of exercise, for example, can contribute to long-term engagement [90,91,92]. While both encoding schemes were marginally motivating, participants felt more competent using Cartesian Endpoint Encoding, and this encoding scheme demonstrated acceptable utility. These findings suggest that users may be more likely to adopt Cartesian Endpoint Encoding for long-term use in a system designed to enhance movement quality by providing real-time supplemental vibrotactile kinesthetic feedback.

### 4.2. Limitations and Future Directions

The limitations of this study are important to consider. The vibrotactile display was fixed to the nonmoving arm using athletic tape to secure the eccentric rotating mass motors against the skin. Although the study team attempted to apply consistent pressure when applying the vibrotactile display, we did not implement an effective way to measure and control the applied pressure. A future implementation of the vibrotactile display could embed the motors in an elastic sleeve (see, for example, [14,46,93]), which could facilitate consistency in the placement and setup of the vibrotactile display across don/doff cycles and across participants. Another limitation stems from the fact that all participants in this study were under 28 years old. Research has shown that the acuity of intensity discrimination for vibrotactile feedback declines somewhat in older adults [94], although this may be counteracted with long-term training [95], which would be a natural byproduct of long-term use of the system. Another limitation results from the fact that this study only implemented a two-dimensional workspace using a hand-held device that constrained motions to the horizontal plane. Behaviors limited to two dimensions do not fully capture the complexity of real-world interactions with objects in three dimensions. Although it would be simple to implement, adding another pair of motors corresponding to a third dimension of vibrotactile feedback could increase cognitive loading beyond that described here, further exacerbating the observed decomposition strategy through the phenomenon of masking. In masking, the presence of one vibratory stimulus degrades the acuity with which another vibratory stimulus can be perceived when the stimuli are presented simultaneously or very close in time [52,96]. Consequently, presenting more than one channel of information within a single sensory feedback modality may cause participants to decompose their movements by strategically processing each dimension one at a time. We speculate that it can be possible to avoid decomposition using cross-linked, multimodal sensory stimuli (see [97]), such as vibrotactile, auditory (cf., [98]), and skin-stretch feedback (cf., [99]) to obtain smoother, more efficient movements. Future studies should examine the effects of extended training with supplemental kinesthetic feedback in three-dimensional workspaces to examine the extent to which the technology can support functional interactions with real-world objects. For example, it is yet unclear whether the integration of supplemental feedback into the real-time control of the arm and hand is promoted better by training all three dimensions of feedback from first exposure onward or by gradually introducing the feedback one dimension at a time.

## 5. Conclusions

Although there have been recent advancements in wearable technologies for sensory augmentation, it remains unclear how movement information should be encoded into the supplemental feedback provided by the technology. This study investigated the objective utility and subjective experience of two biologically inspired methods of encoding arm kinematics into supplemental vibrotactile feedback. Cartesian Endpoint Encoding mimicked visual feedback by translating hand position into the vibrotactile feedback in a Cartesian reference frame. Joint Angle Encoding was inspired by intrinsic proprioception and provided information on hand position in a reference frame that encoded shoulder and elbow joint angles. We found that both methods provided objective utility in enhancing reach accuracy in the absence of concurrent visual feedback, with Cartesian Endpoint Encoding yielding a greater reduction in target capture errors compared to Joint Angle Encoding. While both encoding methods had passable user satisfaction, and both were found to be intrinsically motivating, only Cartesian Endpoint Encoding had passable usability as reported on surveys. This work will help advise future studies aiming to evaluate the benefits of continuous supplemental kinesthetic feedback in guiding goal-directed reaches.

## Figures and Tables

**Table 1:** Intrinsic Motivation Inventory (IMI) scores.

	Interest & Enjoyment	Perceived Competence *	Effort & Importance	Pressure & Tension	Value & Usefulness
CEE	4.2 ± 1.2	3.7 ± 1.5	5.5 ± 0.9	2.6 ± 1.1	5.3 ± 1.1
JAE	4.1 ± 1.3	2.5 ± 1.0	5.7 ± 0.9	3.0 ± 1.2	4.6 ± 1.1

CEE: Cartesian Endpoint Encoding; JAE: Joint Angle Encoding; Values represent mean score ± 1 SD. * *p* < 0.01 across encoding schemes.

## Data Availability

De-identified data will be made available upon reasonable request.

## Appendix A

We used the following anthropometric and home position data (Table A1), along with Equations (1a) and (1b), to compute joint angles in real-time when providing joint angle feedback (see also Figure 2 for visualization of these measurements).

**Table A1:** Participant demographics and anthropometric data. Columns 4 and 5 locate the participant’s home position relative to their shoulder. Columns 6 and 7 refer to measurements c and d as defined in Figure 2.

Subject Number	Sex	Age	Y_Home_ to Y_Shoulder_ (cm)	X_Home_ to X_Shoulder_ (cm)	c: Shoulder to Elbow (cm)	d: Elbow to Handle (cm)
1	Male	26	23	9	26	32
2	Male	25	24	8	35	36
3	Female	24	19	10	30	31
4	Male	24	28	8	34	36
5	Female	22	20	9	26	28
6	Male	27	23	8	30	35
7	Female	24	24	10	29	29
8	Male	27	24	8	29	36
9	Female	25	22	8	26	29
10	Male	25	19	10	30	31
11	Female	26	20	7	24	29
12	Female	27	23	10	27	26
13	Female	23	24	9	29	32
14	Male	20	21	17	31	36
15	Female	23	20	9	27	28
